# The Effect of Far-Infrared Therapy on the Peritoneal Expression of Glucose Degradation Products in Diabetic Patients on Peritoneal Dialysis

**DOI:** 10.3390/ijms22073732

**Published:** 2021-04-02

**Authors:** Chia-Ning Chang, Chih-Yuan Niu, Ann Charis Tan, Chia-Hao Chan, Chun-Fan Chen, Tz-Heng Chen, Szu-Yuan Li, Yung-Tai Chen, Fan-Yu Chen, Wen-Sheng Liu, Chih-Ching Lin, Guor-Jien Wei

**Affiliations:** 1 Institute of Food Safety and Health Risk Assessment, National Yang Ming Chiao Tung University, Hsinchu 300, Taiwan; jenny1993450473@gmail.com (C.-N.C.); robertliu2001@yahoo.com (W.-S.L.); 2 Institute of Food Safety and Health Risk Assessment, National Yang-Ming University, Taipei 112, Taiwan; 3 Department of Pharmacy, National Taiwan University Hospital, Taipei 100, Taiwan; 4 School of Medicine, National Yang Ming Chiao Tung University, Hsinchu 300, Taiwan; eternaltruth2008@hotmail.com (C.-Y.N.); b8701004@gmail.com (C.-F.C.); s19401021@gmail.com (T.-H.C.); taiwan0113@gmail.com (S.-Y.L.); ytchen0117@gmail.com (Y.-T.C.); nono007tw@gmail.com (F.-Y.C.); 5 School of Medicine, National Yang-Ming University, Taipei 112, Taiwan; 6 Department of Medicine, Division of Nephrology, Taipei Veterans General Hospital, Taipei 112, Taiwan; actan5@gmail.com (A.C.T.); box033@gmail.com (C.-H.C.); 7 Department of Internal Medicine, Division of Nephrology, National Yang Ming Chiao Tung University Hospital, Yilan 260, Taiwan; 8 Department of Medicine, Division of Nephrology, Taipei Veterans General Hospital Fenglin Branch, Hualien 975, Taiwan; 9 Department of Internal Medicine, Division of Nephrology, Taipei City Hospital Heping Fuyou Branch, Taipei 100, Taiwan; 10 Center for General Education, National Taipei University, Taipei 104, Taiwan; 11 Department of Medicine, Division of Nephrology, Taipei City Hospital Zhongxing Branch, Taipei 103, Taiwan; 12 Metabolomics-Proteomics Research Center, National Yang Ming Chiao Tung University, Hsinchu 300, Taiwan

**Keywords:** glucose degradation products, peritoneal dialysis, far-infrared therapy, diabetes mellitus

## Abstract

Peritoneal dialysis (PD) is a treatment modality for end-stage renal disease (ESRD) patients. Dextrose is a common osmotic agent used in PD solutions and its absorption may exacerbate diabetes mellitus, a common complication of ESRD. PD solutions also contain glucose degradation products (GDPs) that may lead to encapsulating peritoneal sclerosis (EPS), a severe complication of PD. A previous study showed that far-infrared (FIR) therapy improved a patient’s gastrointestinal symptoms due to EPS. Due to limited literature on the matter, this study aims to investigate dialysate GDPs and peritoneal function in diabetic patients on PD. Thirty-one PD patients were enrolled and underwent 40 min of FIR therapy twice daily for six months. We demonstrated the effect of FIR therapy on the following: (1) decrease of methylglyoxal *(p* = 0.02), furfural (*p* = 0.005), and 5-hydroxymethylfurfural (*p* = 0.03), (2) increase of D/D0 glucose ratio (*p* = 0.03), and (3) decrease of potassium levels (*p* = 0.008) in both DM and non-DM patients, as well as (4) maintenance and increase of peritoneal Kt/V in DM and non-DM patients, respectively (*p* = 0.03). FIR therapy is a non-invasive intervention that can decrease dialysate GDPs in PD patients by improving peritoneal transport rate and solute removal clearance, while also maintaining dialysis adequacy.

## 1. Introduction

Peritoneal dialysis (PD) is one of the supportive treatment choices for end-stage renal disease (ESRD) patients. Dextrose is the most commonly used osmotic agent in peritoneal dialysis solutions (PDS). On average, PD patients absorb 60% of dextrose in each exchange. The continuous absorption of dextrose leads to hyperglycemia, exacerbation of diabetes mellitus (DM), hyperlipidemia, obesity, malnutrition, and titration of insulin dose in DM patients [1,2]. Glucose degradation products (GDPs) are produced during heat sterilization of PDS [3,4] and participate in the formation of advanced glycation end products, which are formed by reducing sugars and amino acids. These compounds are considered partly responsible for the bio-incompatibility of PDS, contributing to reduced dialysis function and the pathogenesis of peritoneal membrane fibrosis [5,6]. Previous studies demonstrated the association between the GDPs and AGEs to various diseases, such as the complications of DM [7,8,9], vascular injury [10], Alzheimer’s disease, Parkinson’s disease [11], inflammation [12], and peritoneal dialysis-related complications [13,14,15].

GDPs are reactive aldehydes such as glyoxal, methylglyoxal, 5-hydroxymethylfurfural (HMF), furfural, formaldehyde, acetaldehyde, and glucosone (2-Keto-D-glucose, KDG) that have been found in PDS [15,16,17]. The analytical difficulties to determine the aldehydes are attributed to their properties of being easily volatile, highly reactive, and lacking chromophores for detection by the ultraviolet (UV) detector. To resolve these problems, derivatization of aldehydes with appropriate reagents will make products stable with chromophores that can be detected by the UV detector. 2,4-Dinitrophenylhydrazine (2,4-DNPH) is the most widely used derivatization agent, which has been used for the quantification of aldehydes using high-performance liquid chromatography (HPLC) measurements in combination with the UV detector. This method has been commonly utilized for determining aldehydes in rainwater [18], air [19,20,21], and PDS [22].

The cytotoxic effects of GDPs on peritoneal mesothelial cells during peritoneal dialysis may lead to chronic inflammation, calcification, and fibrosis [23,24,25,26], the most severe manifestation of which is encapsulating peritoneal sclerosis with a reported incidence of 0.7–13 per thousand patient-years [27] and commonly presents with gastrointestinal symptoms such as nausea, vomiting, and bowel obstruction with radiologic or surgical evidence of encapsulation [28]. A case report by Ou et al. showed that far-infrared (FIR) therapy improved a patient’s gastrointestinal symptoms due to encapsulating peritoneal sclerosis [29].

There are three types of infrared, namely near-infrared (wavelength of 0.7–1.4 μm), mid-infrared (wavelength of 1.4–3.0 μm), and far-infrared (wavelength of 3.0–100 μm). FIR can penetrate approximately 4 cm beneath the skin and can make molecules vibrate and rotate, thereby raising the surface temperature of the skin and creating a thermal effect [30]. An in vitro study demonstrated an anti-inflammatory effect on cultured human umbilical vein endothelial cells that had undergone 40 min of FIR therapy via the induction of heme oxygenase-1. FIR radiation also suppressed tumor necrosis factor alpha (TNF-α)-mediated expression of E-selectin, vascular cell adhesion molecule-1, intercellular cell adhesion molecule-1, monocyte chemoattractant protein-1, interleukin-8, and the cytokine-mediated adhesion of monocytes to endothelial cells, while there was no effect on cell viability under 40 min of FIR therapy. Eventually, the anti-inflammatory effect of FIR was also demonstrated in patients receiving hemodialysis [31]. In previous animal model literature, FIR was able to increase skin blood flow in rats [32], increase the expression of arterial endothelial nitric oxide synthase and nitric oxide synthesis in hamsters [33], promote angiogenesis in mice with non-thermal effect [34], and inhibit interleukin-6 and TNF-α activity in mice with peritonitis [35]. FIR has been applied to many clinical therapeutic applications where it can improve nasal stuffiness of allergic rhinitis [36], ameliorate access flow and patency of arteriovenous fistula on hemodialysis patients [37,38], and relieve discomforts of primary dysmenorrhea [39].

Because of limited literature about the effects of FIR on peritoneal function and the concentration of dialysate GDPs in diabetic patients, the purpose of this study is to investigate the effect of FIR therapy on the dialysate concentration of GDPs and clinical data of PD patients with and without DM.

## 2. Results

### 2.1. Patient Characteristics

**Figure 1:**
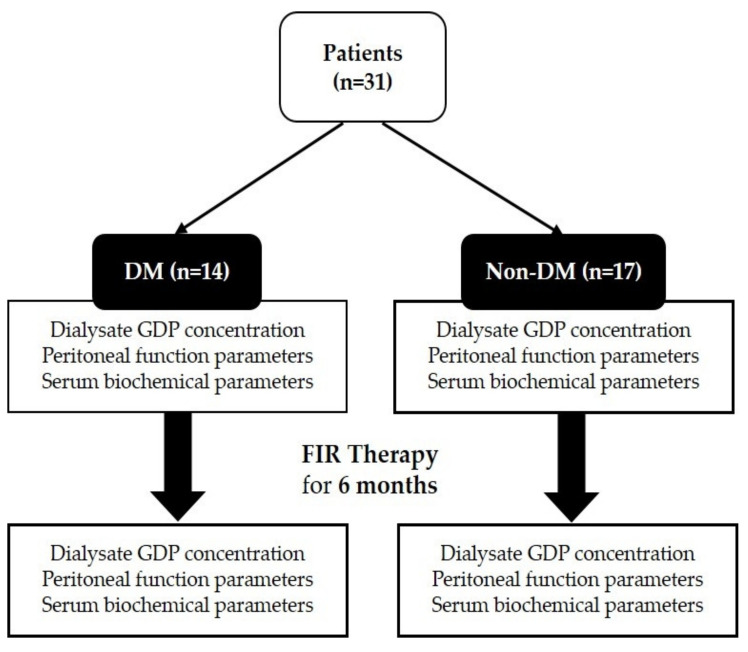
Flow chart of study participants to compare the effect of FIR therapy on DM and non-DM patients. Abbreviations: FIR: far-infrared; DM: diabetes mellitus; GDP: glucose degradation products.

A total of 31 patients enrolled in the study, of which 14 patients had underlying DM (Figure 1). Patients were followed up after six months of FIR therapy. In Table 1, it can be observed that the baseline demographic and clinical parameters between the two groups of patients were similar, except for body mass index (BMI) (*p* = 0.03), glucose (*p* = 0.02), glycated hemoglobin (HbA1c) (*p* = 0.003), and albumin (*p* = 0.001).

### 2.2. Determination of GDPs

**Figure 2:**
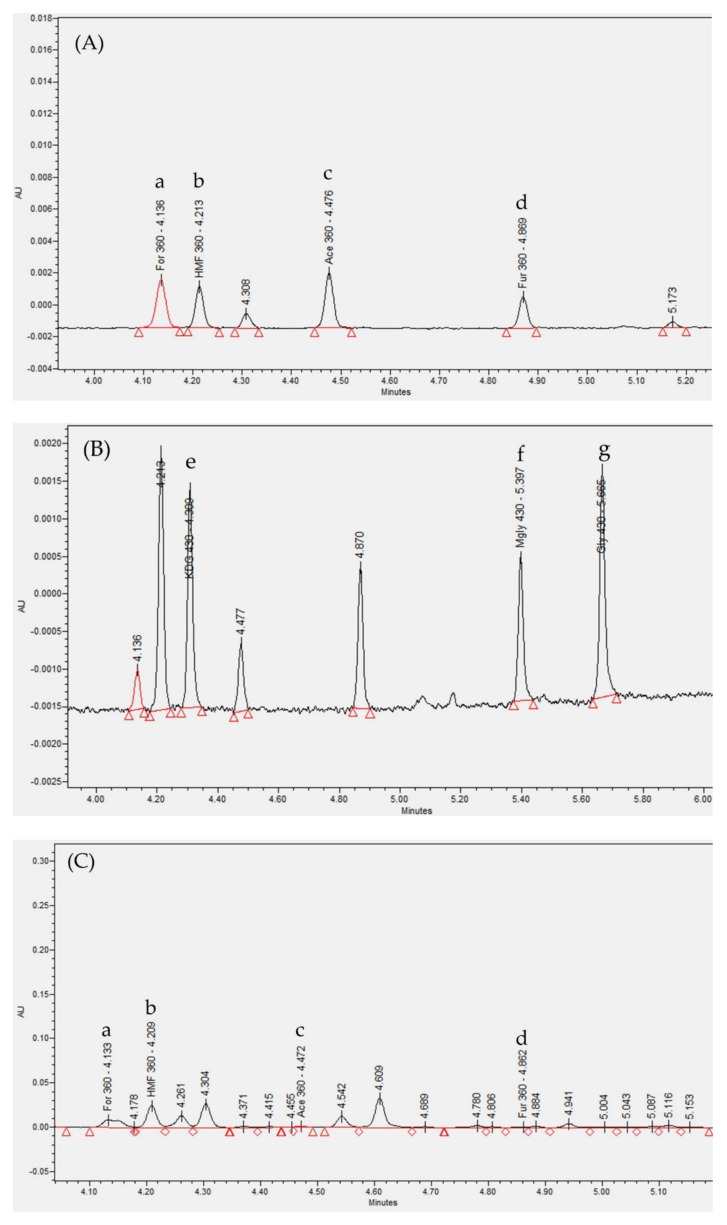

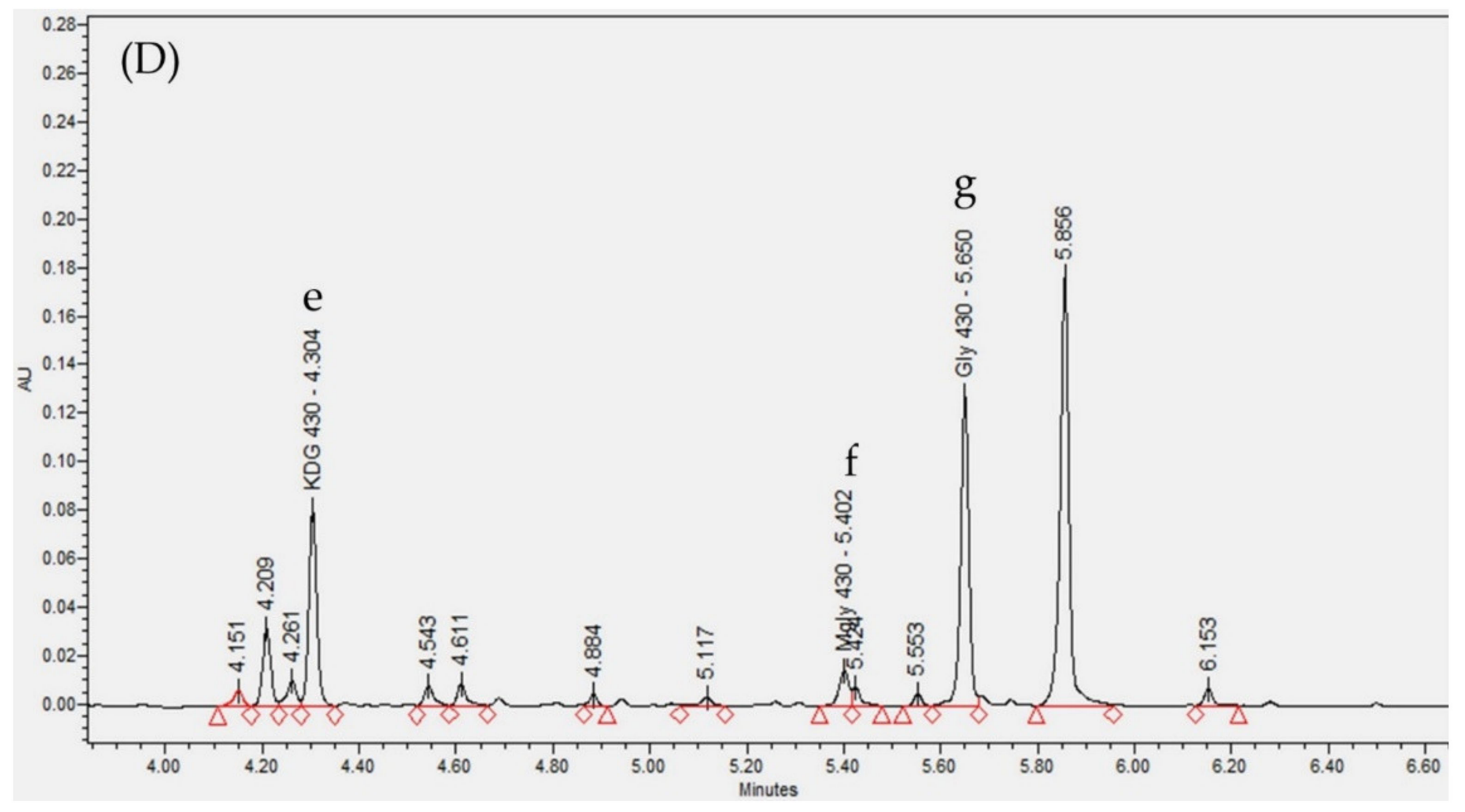
Chromatograms of GDP-DNPH hydrazone derivatives using UPLC-PDA method. The x-axis represents retention time and the y-axis represents UV absorption. (**A**) GDP-DNPH standard solution at UV absorption 360 nm. (**B**) GDP-DNPH standard solution at UV absorption 430 nm. (**C**) GDP-DNPH in dialysate samples at UV absorption 360 nm. (**D**) GDP-DNPH in dialysate samples at UV absorption 430 nm. Abbreviations: GDP: glucose degradation products; DNPH: 2,4-dinitrophenylhydrazine; UPLC-PDA: ultra-performance liquid chromatography photodiode array; UV: ultraviolet.

The chromatograms of GDP-DNPH using the ultra-performance liquid chromatography photodiode array (UPLC-PDA) detector are shown in Figure 2. The identification of GDPs in the dialysate was based on the comparison of UPLC retention time and UV spectra of the standards. Formaldehyde-DNPH (a), HMF-DNPH (b), acetaldehyde-DNPH (c), and furfural-DNPH (d) were detected at 360 nm. KDG-bis-DNPH (e), methylglyoxal-bis-DNPH (f), and glyoxal-bis-DNPH (g) were detected at 430 nm. The linear range and coefficient of determination (r^2^) of each GDP-DNPH hydrazone calibration curve are listed in Table 2. The retention time of the GDP-DNPH in the dialysate corresponded to that of the calibration standard with a tolerance of ±0.1 min [40]. The r^2^ of calibration curves was at least 0.996.

### 2.3. Effect of FIR Therapy

#### 2.3.1. Effect of FIR Therapy on Dialysate GDP Concentration, Peritoneal Function, and Serum Biochemical Parameters

After all the patients underwent FIR therapy, it was observed in Table 3 that three GDPs decreased significantly, namely methylglyoxal (*p* = 0.02), furfural (*p* = 0.005), and HMF (*p* = 0.03). Formaldehyde also exhibited a decreasing trend that approached borderline significance (*p* = 0.06). In terms of clinical parameters, D/D0 glucose ratio increased (*p* = 0.03) while potassium levels decreased (*p* = 0.008). The peritoneal Kt/V of patients increased from 1.69 to 1.82 and approached borderline significance (*p* = 0.09).

#### 2.3.2. Effect of FIR Therapy on Dialysate GDP Concentrations, Peritoneal Function, and Serum Biochemical Parameters in DM and non-DM Patients

In Table 4, no statistical significance in the GDP concentration differences between DM and non-DM patients was observed. However, a decreasing trend can be observed in almost all of the GDPs in both DM and non-DM patients, except for acetaldehyde where an increase can be observed in both groups, though there was a higher incremental change in non-DM patients. Furfural and methylglyoxal demonstrated a 36% and 31% decrease, respectively, post-FIR therapy in DM patients, which was comparable to the 35% and 31% decrease of furfural and methylglyoxal, respectively, seen in non-DM patients. Formaldehyde and glyoxal exhibited a twofold decrease (22% and 27%, respectively) in DM patients in contrast to non-DM patients (12% and 12%, respectively). These values did not reach statistical significance, which may be due to the small number of patients enrolled (n = 31). In Table 5, a significant increase in the peritoneal Kt/V of non-DM patients can be observed post-FIR therapy (*p* = 0.03), while the Kt/V of DM patients remained stable. The pre- and post-FIR therapy GDP concentrations and clinical parameters of DM and non-DM patients can be seen in the supplementary data (Table S1).

## 3. Discussion

Our study demonstrated the statistically significant effect of FIR therapy on the following parameters: (1) decrease of dialysate methylglyoxal (*p* = 0.02), furfural (*p* = 0.005), and HMF (*p* = 0.03), (2) increase of D/D0 glucose ratio (*p* = 0.03), and (3) decrease of serum potassium levels (*p* = 0.008) in both DM and non-DM patients, as well as (4) maintenance and increase of peritoneal Kt/V in DM and non-DM patients, respectively (*p* = 0.03). Even though the GDP concentration differences post-FIR therapy were not significant, a decreasing trend can be observed in almost all the GDPs in both patient groups. Furfural and methylglyoxal demonstrated a 36% and 31% decrease, respectively, in DM patients. Formaldehyde and glyoxal exhibited a twofold decrease in DM patients compared to non-DM patients. A large-scale study should be conducted to further study and confirm the observed trend.

It had been demonstrated that high levels of GDPs and AGEs were related to oxidative stress, cellular damage, and chronic inflammation in humans [3]. At the cellular level, aldehydes cause extensive damage to membrane lipids, cellular proteins, mitochondrial function, RNA and DNA, and disrupt cell signaling [41]. In vitro studies have demonstrated that aldehyde toxicity diminished the viability of frozen mouse oocytes and zygotes, as well as cultured fibroblasts and peritoneal mesothelial cells [42]. Aldehydes interfere with DNA repair mechanisms and have been linked to the development of certain cancers.^7^ Formaldehyde is also recognized as both carcinogenic and mutagenic to humans, based on findings from previous epidemiological, in vivo, and in vitro studies [43]. Tuncer et al. have suggested that PDS containing high acetaldehyde concentrations may be associated with the development of chemical peritonitis in patients [44]. Regarding the short-term cytotoxicity of GDPs on peritoneal mesothelial cells, Witowski et al. conducted a cell proliferation and cell viability study in which human peritoneal cells were exposed to GDPs for 24 h. The results showed that the inhibition of cell proliferation was statistically significant at 1 μg/mL formaldehyde, 10 μg/mL acetaldehyde, methylglyoxal, and glyoxal, and 100 μg/mL furfural [42]. Formaldehyde, glyoxal, methylglyoxal, and furfural were considered to be toxic and reactive GDPs to human peritoneal mesothelial cells [23,24,25,26]. Furfural can irritate exposed mucosa and epithelial cells where acute toxicity included central nervous system depression, lung hemorrhage and congestion, and eye/nasal discharge; and delayed toxicity included hepatic and renal tubular necrosis, hypochromic anemia, and leukopenia [45]. A study noted that dosages of HMF parenteral administration surpassing 75 mg/kg body weight have resulted in some toxic effects in the liver, such as increased hepatic enzyme activity and hepatic fatty degeneration [46]. In our study, methylglyoxal (*p* = 0.02), furfural (*p* = 0.005), and HMF (*p* = 0.03) decreased significantly post-FIR therapy. Since there were limited data on GDP concentration in used PD dialysate, we referred to unused PDS data from previous studies. The highest GDP concentrations found in unused PDS were the following: 18,501 μg/L acetaldehyde (420 μΜ), 450.45 μg/L formaldehyde (15 μΜ), 192.16 μg/L furfural (2 μΜ), 812.56 μg/L glyoxal (14 μΜ), 1657.38 μg/L methylglyoxal (23 μΜ), 3783.3 μg/L HMF (30 μΜ), and 5362.01 μg/L KDG (30.1 μΜ) [16,17]. The dialysate GDP concentrations in our study were considerably higher than the GDP concentrations in unused PD fluids of previous studies. The results may be attributed to PD patients being exposed to exogenous GDPs in their diet and PD fluids, as well as endogenous GDPs from lipid peroxidation and glucose degradation. The dialysate GDP concentrations may represent patients’ excretion of GDPs. Our study results are in agreement to the study conducted by Stinghen et al. where they indicated that there were high levels of AGE excretion in patients on PD [7].

Concerning peritoneal function and serum biochemical parameters post-FIR therapy, D/D0 glucose ratio increased, potassium levels decreased (*p* = 0.008), and peritoneal Kt/V of non-DM patients increased. A high D/D0 glucose ratio can lead to slower glucose absorption into the circulation leading to a higher osmotic gradient across the peritoneal membrane, which may result in a better ultrafiltration effect. This may explain why FIR therapy was able to decrease the concentration of the water-soluble GDPs [28]. It should also be noted that there was a higher mean difference of D/D0 glucose ratio in DM patients than in non-DM patients post-FIR therapy (0.04 vs. 0.02, respectively), which may be the reason why formaldehyde and glyoxal decreased twofold in DM patients. Diabetic patients often have reduced renal function to excrete potassium, thereby having an increased risk of hyperkalemia [47]. After undergoing FIR therapy, the potassium levels of patients decreased (*p* = 0.008), which may be due to the increased ultrafiltration capacity attributed to the high D/D0 glucose ratio observed. The peritoneal Kt/V in non-DM patients increased post-FIR therapy, while the Kt/V of DM patients remained stable, where dialysis adequacy was maintained in both groups of patients after undergoing treatment. Based on the observed trends, the effect of FIR therapy in decreasing GDP concentrations, lowering potassium levels, and improving dialysis adequacy is worth evaluating in future studies with a larger sample size to confirm the results in this study.

There were several limitations in our study, namely the study being conducted in a single center in Taiwan, had a small sample, and had no control group (have not undergone FIR therapy) to compare with. Future studies should be conducted to confirm the effect of FIR therapy on dialysate GDPs and the clinical status of PD patients that we have seen in this initial study. Despite these limitations, this study is the first in vivo human study that demonstrated the effect of an intervention, specifically FIR therapy, on dialysate GDP concentrations and peritoneal function. The detection method used to detect the GDPs in used PDS with a high dextrose concentration is also very stable and the first to be conducted.

## 4. Materials and Methods

### 4.1. Study Design

This study is a prospective analysis conducted in a single center and was approved by the Institutional Review Board of Taipei Veterans General Hospital. The participants were recruited from the Taipei Veterans General Hospital peritoneal dialysis outpatient department from November 25, 2016 to September 5, 2018. We included the patients who met the following criteria: (1) ESRD patients aged 20–90 years without receiving FIR therapy within 12 months; (2) receiving continuous ambulatory peritoneal dialysis or automated peritoneal dialysis; (3) no history of peritonitis, cerebrovascular accident, myocardial infarction, or receiving any cardiovascular intervention in the past 3 months. After informed consent was obtained, patients were allocated to two groups based on their underlying DM history. Both groups of PD patients received FIR therapy for 6 months. The demographic and clinical data of the patients were recorded from the hospital’s electronic database. We collected the last daily bag of peritoneal dialysate and compared the dialysate concentration of GDPs and clinical data in PD patients pre- and post-FIR therapy.

The demographic data of the patients were comprised of their age, gender, weight (kg), BMI (kg/m^2^), PD duration (months), daily exposure of dextrose from PD fluids (g/24 hr), underlying causes of ESRD, comorbidities, and medications. The clinical data collected included peritoneal function and serum biochemical parameters. The peritoneal function parameters were comprised of D/D0 glucose (ratio of dialysate glucose after time of dwell to initial dialysate glucose) and D/P creatinine (dialysate/plasma creatinine ratio at 4 h), peritoneal Kt/V urea, peritoneal weekly creatinine clearance (CCr, L/wk/1.73 m^2^), net ultrafiltration (mL), normalized protein catabolic rate (nPCR, g/kg/day), and urine output (mL). The serum biochemical parameters were comprised of fasting blood glucose (mg/dL), HbA1c (%), triglyceride (mg/dL), albumin (g/dL), potassium (K, mmol/L), and high-sensitivity C-reactive protein (hs-CRP, mg/dL). The GDPs included in the study were glyoxal, methylglyoxal, HMF, furfural, formaldehyde, acetaldehyde, and KDG. The dialysate concentration of GDPs was analyzed by the UPLC-PDA detector.

### 4.2. Far-Infrared Therapy

**Figure 3:**
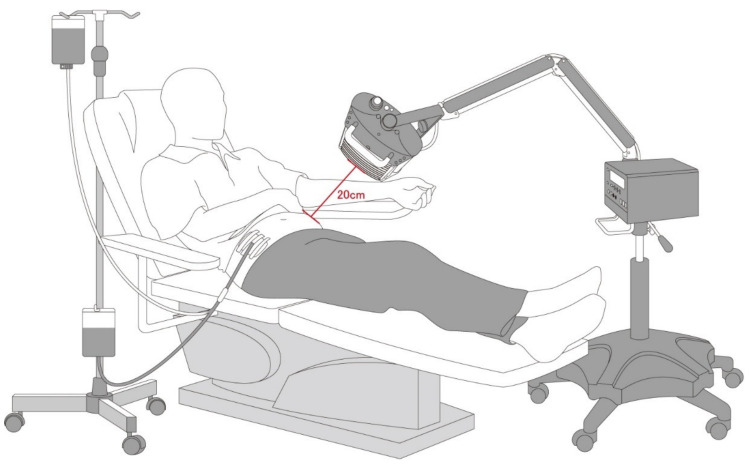
FIR therapy set-up during a PD exchange. Abbreviation: FIR: far-infrared; PD: peritoneal dialysis.

The WS TY101 FIR emitter (WS Far Infrared Medical Technology Co., Ltd., Taipei, Taiwan) was used for the intervention in this study. The electrified ceramic plates of this emitter generate electromagnetic waves with wavelengths in the range of 3–25 μm. The irradiating power density was 20 mW/cm^2^ and the top radiator was set at a height of 20 cm above the surface of the abdomen (Figure 3). The patients underwent FIR therapy for 40 min twice daily during the first and last exchange for 6 months.

### 4.3. Determination of GDPs

#### 4.3.1. Reagents and Chemicals

2,4-dinitrophenylhydrazine (2,4-DNPH, 97%, reagent grade) was purchased from Sigma-Aldrich and using as a derivatization agent. Tetrahydrofuran (ACS grade) was purchased from Macron Fine Chemicals™. Ethanol (95% v/v) was purchased from Echo Chemical. Acetonitrile (HPLC grade) was purchased from J. T. Baker^®^. Formic acid (FA, for mass spectrometry, ~98%) was purchased from Honeywell Fluka™. Hydrochloric acid (37% w/w) was purchased from Fisher Chemical. Deionized water was double distilled and filtered by Milli-Q SP Reagent Water system. Formaldehyde-2,4-DNPH (>98%) and acetaldehyde-2,4-DNPH (>98%) were purchased from Tokyo Chemical Industry. Furfural (99%), glyoxal (40% w/w aqueous solution), methylglyoxal (40% w/w aqueous solution), 5-hydroxymethyl-2-furaldehyde (HMF, 99%), and 2-Keto-D-glucose (KDG, ≥98%) were purchased from Sigma-Aldrich. The stock solution (100 mg/L) of all aldehyde-DNPH were prepared in tetrahydrofuran and stored at −20 °C.

The last daily bag of PDS included dianeal PD-2 PDS with 1.5% dextrose, dianeal PD-2 PDS with 2.5% dextrose, dianeal low calcium (2.5 mEq/L) PDS with 1.5% dextrose, dianeal low calcium (2.5 mEq/L) PDS with 2.5% dextrose, and extraneal PDS with 7.5% icodextrin, were all sourced from Baxter Healthcare SA, Singapore Branch. Balance 2.3% glucose (1.75 mmol/L calcium) PDS was from Fresenius Medical Care, Germany. The patients’ last daily bag PD prescriptions were unchanged pre- and post-FIR therapy.

#### 4.3.2. Derivatization Process of Standards

All aldehydes were identified as hydrazone derivatives using 2,4-DNPH (DNPH) as a reagent. The standards of furfural and HMF hydrazone derivatives were synthesized by reacting an excess of the selected carbonyl compounds, which were dissolved in 95% v/v ethanol with 2,4-DNPH, and few drops hydrochloric acid was added as a catalyst. Bicarbonyl compounds, including glyoxal, methylglyoxal, and KDG hydrazone derivatives, were synthesized by reacting with an excess of 2,4-DNPH. Finally, the precipitate products were filtered off, recrystallized from hot tetrahydrofuran, washed with cold water, and dried in an oven. The purity of the products was identified by UPLC-PDA and no impurity peaks were detected.

#### 4.3.3. Instruments

The UPLC-PDA system used the Waters ACQUITY UPLC I-class plus system with the ACQUITY UPLC PDA eλ detector. An ACQUITY UPLC^®^HSS C18 SB column (1.8 μm, 2.1 mm × 100 mm) equipped with an ACQUITY UPLC^®^HSS C18 VanGuard^TM^ pre-column (1.8 μm, 2.1 mm × 5 mm) was used for the separation. The column oven was set at 30 °C. The PDA detection wavelength range was from 200 to 780 nm. Formaldehyde-DNPH, acetaldehyde-DNPH, furfural-DNPH, and HMF-DNPH were detected at 360 nm. Glyoxal-bis-DNPH, methylglyoxal-bis-DNPH, and KDG-bis-DNPH were detected at 430 nm. The mobile phase containing 0.1% formic acid in water was designated as mobile phase A and acetonitrile containing 0.1% FA was designated as mobile phase B. The gradient mode was as follows: 0–0.5 min, 5% B; 0.5–7 min, 5%–100% B; 7–8 min, 100% B min; 8–8.5 min, 100%–5% B; and 8.5–9 min, 5% B. The flow rate was 0.6 mL/min and the injection volume was 5 μL. Data acquisition and processing were carried out by Empower 3 software.

#### 4.3.4. Dialysate Sample Preparations

DNPH solution was prepared as 1 mM DNPH in acetonitrile with 0.5 N hydrochloric acid. 10 μL of dialysate sample was mixed with 1000 μL of DNPH solution, vortexed 2 min, and then shaken for at least 12 h by an orbital shaker (50 rpm). All samples were centrifuged the next day at 13,000 rpm for 15 min using HSIANGTAI Centrifuge, after which the supernatant was collected. All sample supernatants were filtered using a Supelco^®^ 96-well protein precipitation filter plate. After filtering, all sample mixtures were dried under nitrogen and reconstituted in 200 μL of 50% acetonitrile containing 0.1% FA.

#### 4.3.5. Quantification of GDPs

The quantification was carried out with calibration curves using the peak area of GDPs. For the calibration curves, all stock solution (100 mg/L GDP-DNPH) were prepared and diluted with 50% acetonitrile containing 0.1% FA to standard solutions of 25 μg/L, 50 μg/L, 100 μg/L, 250 μg/L, 500 μg/L, 1 mg/L, 2.5 mg/L, 5 mg/L, and 10 mg/L. The acquisition calibration curves and the concentration of GDP-DNPH using Empower 3 with weighted least squares method and weighting factor are 1/x. Formaldehyde-DNPH, acetaldehyde-DNPH, furfural-DNPH, and HMF-DNPH were determined at 360 nm. Glyoxal-bis-DNPH, methylglyoxal-bis-DNPH, and KDG-bis-DNPH were determined at 430 nm. GDP data were calculated after unit conversion from GDP-DNPH using Microsoft Excel.

### 4.4. Statistical Analysis

Data were analyzed using SPSS Statistics, version 22. Continuous variables were presented as mean and standard deviation for paired *t*-test (two-tailed). Mann–Whitney U test and Wilcoxon signed-rank test (two-tailed) were used for nonparametric statistical analysis. Categorical variables were presented as number and percentage for Fisher’s exact test. *p* < 0.05 was considered statistically significant.

## 5. Conclusions

In conclusion, our study demonstrated that FIR therapy can decrease PD patients’ dialysate GDPs by improving peritoneal transport rate and solute removal clearance, while also maintaining dialysis adequacy.

## Figures and Tables

**Table 1:** Baseline demographic and clinical parameters of DM and non-DM patients.

Parameters	DM (n = 14)	Non-DM (n = 17)	*p*
Age (years)	59.5 ± 12.11	55.71 ± 14.29	0.48
Gender			
Male	6 (42.9)	6 (35.3)	0.72
Female	8 (57.1)	11 (64.7)	
Weight (kg)	61.32 ± 7.21	57.89 ± 12.66	0.25
BMI (kg/m^2^)	24.16 ± 2.60	21.60 ± 3.75	0.03 *
PD duration (months)	14.07 ± 16.91	25.65 ± 31.82	0.34
Peritoneal fluid dextrose exposure (g/24 h)	113.29 ± 35.55	116.10 ± 38.95	0.75
Comorbidities			
Hypertension	14 (100)	14 (82.4)	0.23
Hyperlipidemia	4 (28.6)	9 (52.9)	0.28
Congestive heart failure	3 (21.4)	3 (17.6)	1.0
Gout	3 (21.4)	8 (47.1)	0.26
Medications			
Angiotensin receptor blockers	8 (57.1)	7 (41.2)	0.48
Beta blockers	7 (50)	9 (52.9)	1.0
HMG-CoA reductase inhibitors	8 (57.1)	12 (70.6)	0.48
Peritoneal function			
D/D0 glucose	0.38 ± 0.07	0.35 ± 0.08	0.23
D/P creatinine	0.67 ± 0.11	0.69 ± 0.10	0.34
Peritoneal Kt/V	1.78 ± 0.42	1.62 ± 0.33	0.31
Peritoneal weekly CCr (L/week/1.73 m^2^)	42.92 ± 7.97	39.56 ± 8.40	0.30
Net ultrafiltration (mL)	839.93 ± 564.45	1007 ± 517.64	0.51
Urine output (mL)	355 ± 298.19	523.82 ± 685.16	0.90
nPCR (g/kg/day)	1.02 ± 0.22	1.19 ± 0.21	0.06
Serum biochemistry			
Glucose (mg/dL)	136 ± 60.52	102.53 ± 13.45	0.02 *
HbA1c (%)	6.85 ± 1.21	5.57 ± 0.57	0.003 **
Triglycerides (mg/dL)	135.21 ± 79.25	124.47 ± 49.91	0.89
BUN (mg/dL)	74.29 ± 22.32	75.59 ± 14.08	0.50
Creatinine (mg/dL)	9.97 ± 2.78	11.88 ± 3.34	0.15
Albumin (g/dL)	3.25 ± 0.33	3.77 ± 0.43	0.001 **
Potassium (mmol/L)	4.24 ± 0.86	4.12 ± 0.47	0.84
Calcium (mg/dL)	9.72 ± 0.66	9.55 ± 0.52	0.39
Phosphate (mg/dL)	4.81 ± 1.27	5.32 ± 1.29	0.29
Uric acid (mg/dL)	5.16 ± 1.50	5.99 ± 1.36	0.09
Hs-CRP (mg/dL)	2.07 ± 5.22	0.36 ± 0.63	0.11

Continuous variables were presented as mean ± standard deviation. Categorical variables were presented as number (percentage). *: *p* < 0.05; **: *p* < 0.01 by using Mann–Whitney U test (two-tailed)/Fisher’s exact test. Abbreviations: DM: diabetes mellitus; BMI: Body Mass Index; PD: peritoneal dialysis; ESRD: end-stage renal disease; HMG-CoA: hydroxymethylglutaryl-CoA; D/D0 glucose: ratio of dialysate glucose after time of dwell to initial dialysate glucose; D/P creatinine: dialysate/plasma creatinine ratio at 4 h; Kt/V: Kt/V urea; CCr: creatinine clearance; nPCR: normalized protein catabolic rate; HbA1c: glycated hemoglobin; BUN: blood urea nitrogen; hs-CRP: high-sensitivity C-reactive protein.

**Table 2:** Linear range of UPLC-PDA calibration curve for dialysate GDP-DNPH compounds.

GDP-DNPH Compounds	Linear Range	r^2^
Formaldehyde-DNPH	100 μg/L–5 mg/L	0.997
Acetaldehyde-DNPH	25 μg/L–1 mg/L	0.996
Furfural-DNPH	10 μg/L–500 μg/L	0.998
HMF-DNPH	250 μg/L–10 mg/L	0.998
Glyoxal-bis-DNPH	250 μg/L–10 mg/L	0.998
Methylglyoxal-bis-DNPH	50 μg/L–2.5 mg/L	0.997
KDG-bis-DNPH	250 μg/L–10 mg/L	0.998

Abbreviations: UPLC-PDA: ultra-performance liquid chromatography photodiode array; GDP: glucose degradation products; DNPH: 2,4-dinitrophenylhydrazine; r^2^: coefficient of determination; HMF: 5-hydroxymethyl-2-furaldehyde; 2-Keto-D-glucose: KDG.

**Table 3:** Comparison of dialysate GDP concentrations and clinical parameters pre-and post-FIR therapy.

Parameters	Pre-FIR	Post-FIR	*p*
GDPs (μg/L)			
Formaldehyde	4070.94 ± 1764.60	3362.85 ± 1233.10	0.06
Acetaldehyde	1811.45 ± 388.50	1909.26 ± 415.32	0.34
Furfural	1366.25 ± 726.71	876.11 ± 491.88	0.005 **
HMF	51,514.0266 ± 37,816.87	35,565.57 ± 25,674.87	0.03 *
Glyoxal	7025.46 ± 3137.69	5713.31 ± 4798.52	0.20
Methylglyoxal	2260.49 ± 1497.04	1550.11 ± 1329.83	0.02 *
KDG	33,879.82 ± 15,265.64	29,031.18 ± 16,173.89	0.18
Peritoneal function			
D/D0 glucose	0.37 ± 0.08	0.39 ± 0.06	0.03 *
D/P creatinine	0.68 ± 0.11	0.66 ± 0.08	0.15
Peritoneal Kt/V	1.69 ± 0.37	1.82 ± 0.39	0.09
Peritoneal weekly CCr (L/week/1.73 m^2^)	41.07 ± 8.25	41.69 ± 7.54	0.70
nPCR (g/kg/d)	1.11 ± 0.23	1.12 ± 0.30	0.89
Serum biochemistry			
Glucose (mg/dL)	117.65 ± 44.39	132.19 ± 53.33	0.12
HbA1c (%)	6.14 ± 1.11	6.32 ± 1.21	0.25
Triglycerides (mg/dL)	127.5 ± 64.13	155.43 ± 111.97	0.12
Albumin (g/dL)	3.54 ± 0.46	3.49 ± 0.48	0.44
Potassium (mmol/L)	4.17 ± 0.67	3.81 ± 0.66	0.008 **
Hs-CRP (mg/dL)	1.13 ± 3.57	0.48 ± 0.82	0.23

Continuous variables were presented as mean ± standard deviation for paired *t*-test (two-tailed). *: *p* < 0.05; **: *p* < 0.01 by using paired *t*-test (two-tailed). Abbreviations: GDP: glucose degradation products; FIR: far-infrared; HMF: 5-hydroxymethyl-2-furaldehyde; 2-Keto-D-glucose: KDG; D/D0 glucose: ratio of dialysate glucose after time of dwell to initial dialysate glucose; D/P creatinine: dialysate/plasma creatinine ratio at 4 h; Kt/V: Kt/V urea; CCr: creatinine clearance; nPCR: normalized protein catabolic rate; HbA1c: glycated hemoglobin; hs-CRP: high-sensitivity C-reactive protein.

**Table 4:** Comparison of the differences in dialysate GDP concentration post-FIR therapy in DM and non-DM patients.

Δ GDPs (μg/L)	DM	*p* ^†^	Non-DM	*p* ^†^	*p* ^††^
Formaldehyde	−1082.07 −22.33)	0.16	−400.10 (−11.65)	0.23	0.5
Acetaldehyde	5.98 (0.32)	0.83	173.44 (9.93)	0.25	0.34
Furfural	−534.10 −36.40)	0.08	−453.94 (−35.38)	0.11	0.81
HMF	−13,822.86 (−24.54)	0.16	−17,698.96 (−37.22)	0.16	1.0
Glyoxal	−1909 (−27.14)	0.16	−820.62 (−11.69)	0.59	0.43
Methylglyoxal	−698.38 (−31.43)	0.11	−720.26 (−31.42)	0.15	1.0
KDG	−6543.83 (−17.01)	0.51	−3452.59 (−11.47)	0.29	0.81

Δ = Post-FIR values–Pre-FIR values. Continuous variables were presented as mean difference (percentage difference). ^†^ *p* between pre- and post-FIR therapy values were calculated by using Wilcoxon signed-rank test (two-tailed). ^††^ *p* between DM and non-DM values were calculated by using Mann–Whitney U test (two-tailed). Abbreviations: GDP: glucose degradation products; FIR: far-infrared; DM: diabetes mellitus; HMF: 5-hydroxymethyl-2-furaldehyde; 2-Keto-D-glucose: KDG.

**Table 5:** Comparison of the differences in clinical parameters post-FIR therapy in DM and non-DM patients.

Δ Clinical Parameters	DM	Non-DM	*p*
Peritoneal function			
D/D0 glucose	0.04 ± 0.09	0.02 ± 0.04	0.77
D/P creatinine	−0.007 ± 0.10	−0.03 ± 0.06	0.36
Peritoneal Kt/V	−0.04 ± 0.24	0.27 ± 0.47	0.03 *
Peritoneal weekly CCr (L/week/1.73 m^2^)	−1.63 ± 8.14	2.46 ± 9.08	0.22
nPCR (g/kg/d)	0.003 ± 0.22	0.008 ± 0.22	0.85
Serum biochemistry			
Glucose (mg/dL)	33.64 ± 70.05	−1.18 ± 11.58	0.44
HbA1c (%)	0.24 ± 1.15	0.12 ± 0.46	0.74
Albumin (g/dL)	−0.01 ± 0.38	−0.08 ± 0.31	0.59
Potassium (mmol/L)	−0.56 ± 0.61	−0.20 ± 0.77	0.20
Hs-CRP (mg/dL)	−1.32 ± 4.37	−0.1 ± 0.65	0.61

Δ = Post-FIR values–Pre-FIR values. Continuous variables were presented as mean difference ± standard deviation. *: *p* < 0.05; by using Mann–Whitney U test (two-tailed). Abbreviations: FIR: far-infrared; DM: diabetes mellitus; D/D0 glucose: ratio of dialysate glucose after time of dwell to initial dialysate glucose; D/P creatinine: dialysate/plasma creatinine ratio at 4 h; Kt/V: Kt/V urea; CCr: creatinine clearance; nPCR: normalized protein catabolic rate; HbA1c: glycated hemoglobin; hs-CRP: high-sensitivity C-reactive protein.

## Data Availability

Not applicable.

## Supplementary Materials

The following are available online at https://www.mdpi.com/article/10.3390/ijms22073732/s1.
