# Supplementary material for: The Effect of Far-Infrared Therapy on the Peritoneal Expression of Glucose Degradation Products in Diabetic Patients on Peritoneal Dialysis
Source: Int J Mol Sci. 2021 Apr 2;22(7):3732. doi: 10.3390/ijms22073732 (PMC8038268; doi:10.3390/ijms22073732)
Supplement: Supplementary file 1 [file ijms-22-03732-s001.pdf]

Table S1. Comparison of dialysate GDP concentrations and clinical parameters pre- and post-FIR therapy in DM and non-DM patients

Parameters	DM		*P*	Non-DM		*P*
	Pre-FIR	Post-FIR		Pre-FIR	Post-FIR	
GDPs (μg/L)						
Formaldehyde	4845.68±2034.39	3763.61±1516.77	0.16	3432.91±1232.04	3032.81±851.77	0.23
Acetaldehyde	1889.60±438.37	1895.58±396.97	0.83	1747.09±342.21	1920.53±441.68	0.25
Furfural	1467.19±685.11	933.09±440.95	0.08	1283.12±769.79	829.18±538.97	0.11
HMF	56,317.52±39711.71	42,494.66±32097.18	0.16	47,558.21±36923.19	29,859.25±17953.52	0.16
Glyoxal	7034.68±3121.20	5125.68±5003.88	0.16	7017.86±3247.17	6197.24±4720.01	0.59
Methylglyoxal	2222.19±1499.73	1523.81±1460.20	0.11	2292.04±1540.23	1571.78±1257.92	0.15
KDG	38,460.35±17028.53	31,916.52±19623.39	0.51	30,107.61±12956.49	26,655.02±12819.17	0.29
Peritoneal function						
D/D0 glucose	0.38±0.07	0.42±0.06	0.24	0.35±0.08	0.37±0.06	0.03*
D/P creatinine	0.67±0.11	0.66±0.08	0.88	0.69±0.10	0.66±0.09	0.04*
Peritoneal Kt/V	1.78±0.42	1.74±0.35	0.71	1.62±0.33	1.89±0.42	0.007**
Peritoneal weekly CCr (L/week/1.73 m^2)	42.92±7.97	41.29±5.21	0.60	39.56±8.40	42.02±9.17	0.21
nPCR (g/kg/d)	1.02±0.22	1.03±0.29	0.83	1.19±0.21	1.20±0.29	0.91
Serum biochemistry						
Glucose (mg/dL)	136±60.52	169.64±60.15	0.22	102.53±13.45	101.35±12.44	0.80
HbA1c (%)	6.85±1.21	7.09±1.28	0.47	5.57±0.57	5.69±0.70	0.30
Albumin (g/dL)	3.25±0.33	3.24±0.44	0.92	3.77±0.43	3.69±0.41	0.31
Potassium (mmol/L)	4.24±0.86	3.68±0.54	0.004**	4.12±0.47	3.92±0.75	0.30
Hs-CRP (mg/dL)	2.07±5.22	0.75±1.14	0.55	0.36±0.63	0.26±0.31	0.48

Continuous variables were presented as mean ± standard deviation.

*: $P < 0.05$; **: $P < 0.01$ by using Wilcoxon signed-rank test (two-tailed).

Abbreviations: GDP: glucose degradation products; FIR: far-infrared; DM: diabetes mellitus; D/D0 glucose: ratio of dialysate glucose after time of dwell to initial dialysate glucose; D/P creatinine: dialysate/plasma creatinine ratio at 4 h; Kt/V: Kt/V urea; CCr: creatinine clearance; nPCR: normalized protein catabolic rate; HbA1c: glycated hemoglobin; hs-CRP: high-sensitivity C-reactive protein.
